# Early Stage Root-Associated Fungi Show a High Temporal Turnover, but Are Independent of Beech Progeny

**DOI:** 10.3390/microorganisms8020210

**Published:** 2020-02-04

**Authors:** Kezia Goldmann, Silke Ammerschubert, Rodica Pena, Andrea Polle, Bin-Wei Wu, Tesfaye Wubet, François Buscot

**Affiliations:** 1 UFZ-Helmholtz-Centre for Environmental Research, Department of Soil Ecology, Theodor-Lieser-Straße 4, 06120 Halle (Saale), Germany; francois.buscot@ufz.de; 2 Forest Botany and Tree Physiology, University of Goettingen, Büsgenweg 2, 37077 Göttingen, Germany; sammers@gwdg.de (S.A.); Rodica.Pena@forst.uni-goettingen.de (R.P.); apolle@gwdg.de (A.P.); 3 Chinese Academy of Sciences, Institute of Microbiology, State Key Laboratory of Mycology, 1 Beichen West Road, Chaoyang District, Beijing 100101, China; wubinwei@163.com; 4 UFZ-Helmholtz-Centre for Environmental Research, Department of Community Ecology, Theodor-Lieser-Straße 4, 06120 Halle (Saale), Germany; tesfaye.wubet@ufz.de; 5 German Centre for Integrative Biodiversity Research (iDiv) Halle-Jena-Leipzig, Deutscher Platz 5e, 04103 Leipzig, Germany

**Keywords:** *Fagus sylvatica*, rhizosphere, fungal ITS2, Illumina sequencing, *Beech Transplant Experiment*, Biodiversity Exploratories

## Abstract

The relationship between trees and root-associated fungal communities is complex. By specific root deposits and other signal cues, different tree species are able to attract divergent sets of fungal species. Plant intraspecific differences can lead to variable fungal patterns in the root’s proximity. Therefore, within the *Beech Transplant Experiment*, we analyzed the impact of three different European beech ecotypes on the fungal communities in roots and the surrounding rhizosphere soil at two time points. Beech nuts were collected in three German sites in 2011. After one year, seedlings of the different progenies were out-planted on one site and eventually re-sampled in 2014 and 2017. We applied high-throughput sequencing of the fungal ITS2 to determine the correlation between tree progeny, a possible home-field advantage, plant development and root-associated fungal guilds under field conditions. Our result showed no effect of beech progeny on either fungal OTU richness or fungal community structure. However, over time the fungal OTU richness in roots increased and the fungal communities changed significantly, also in rhizosphere. In both plant compartments, the fungal communities displayed a high temporal turnover, indicating a permanent development and functional adaption of the root mycobiome of young beeches.

## 1. Introduction

Unravelling the relationship between plants and soil microbes is still one of the challenges in ecology. The importance of soil microorganisms to support plants is well established [1]. Likewise, it is known that plants shape the soil microbial diversity by providing different kinds of exudates and litter [2]. Moreover, it was previously shown that both the plant species identity and intraspecific plant diversity can affect the associated microbiomes [3,4,5]. Besides seasonal differences in microbial diversity and activity in soils [6,7,8], the plant-development stage led to variability in associated microbiomes [9].

Due to their longevity, trees have to adapt constantly to changing environmental conditions, and thus are characterized by divergent microbial assemblies intra- and inter-annually [10,11,12,13,14]. Exchange between microbes and plants often occurs in the rhizosphere [15], and is mediated by an interplay of tree age-related root exudates and various microbial deposits [16]. However, temporal studies on the role of host ecotype in shaping the soil microbiome under field conditions are still scarce [17,18,19]. Though, it would be interesting to understand the microbial interaction with temperate forest trees, since they are characterized by a low level of tree species diversity [20]. European beech (*Fagus sylvatica* L.) is the most dominant native tree in Central Europe and is characterized by a high plasticity [21]. Beech trees are known for their local adaptation [22]. Hence, different beech ecotypes are ideal to study associated microbes and to determine whether a “home-field advantage” (HFA) occurs. HFA suggests that, for example, the microbial decomposition of litter or deadwood is increased in original habitats (i.e., home) compared to distant habitats with differing plant communities (i.e., away from home) [23,24]. Likewise, we suggest that there is an increased ability of plant acclimatization and adaptation to their habitat of origin due to recruitment of a locally adapted specific microbiome, characterized by an increase of mutualistic symbionts and reduction of pathotrophs.

Amongst soil microorganisms, fungi are engaged in especially close relationships with plants and react sensitive to vegetation shifts [25,26]. These fungi can occur as mutualist (e.g., mycorrhiza) or detrimental plant partners (e.g., pathogens and parasites) [27]. Since plant residues are the dominant part of dead organic matter, fungal saprotrophs are also plant-dependent, e.g., deadwood fungi display high substrate specificity [28]. In forests, trees are dominant and accordingly, the abundance of ectomycorrhizal fungi (EMF) is increased [16,29]. Through complex belowground networks, fungal mycelium connects multiple trees and understory plants, which allows the transport of nutrients across trophic levels [29]. Saprotrophic fungi, found in litter and upper soil layers, are likewise important in forest ecosystems [16]. They are involved in the majority of decomposition processes, leading to release and availability of nutrients [30]. Within the root proximity, saprotrophic fungi realize the immediate decomposition of dead roots or deceased microbes [31].

The *Beech Transplant Experiment* (BTE) was designed to understand how different beech progenies impact the rhizosphere and root mycobiomes at early tree development stages. As part of the *Biodiversity Exploratories* project [32], BTE profits from the overall design: Beech nuts were collected in 2011 on beech-dominated experimental plots (EPs) at three sites along a north–south gradient across Germany. These sites differ in topography as well as soil characteristic and climatic conditions [32,33]. Accordingly, the beech nuts collected at individual sites are considered as progenies of different beech ecotypes [34]. After cultivation in the greenhouse in neutral soil, beech seedlings were out-planted in 2012 to beech dominated EPs in Schorfheide-Chorin, the *Exploratory* in northern Germany. In 2014 and 2017, roots and rhizosphere soils were sampled and used for molecular analyses of the fungal communities. For this, the fungal internal transcribed spacer (ITS2) region was Illumina sequenced. We aimed to study the fungal turnover after three years during this early stage of plant development. Since beech progenies from Schorfheide-Chorin may have a home-field advantage, we included also progenies from the two other *Exploratories* Schwäbische Alb and Hainich-Dün. We hypothesized that 1) rhizosphere and root fungal communities are influenced by beech progeny; 2) thereby, the beeches originating from Schorfheide-Chorin have a HFA, characterized by a higher number of symbiotic fungal partners coupled with a reduction of pathotrophs; 3) due to on-going beech development, we expect the fungal richness to be temporally variable and linked to high fungal community turnover rates.

## 2. Materials and Methods

### 2.1. Study Sites and Experimental Design

**Figure 1:**
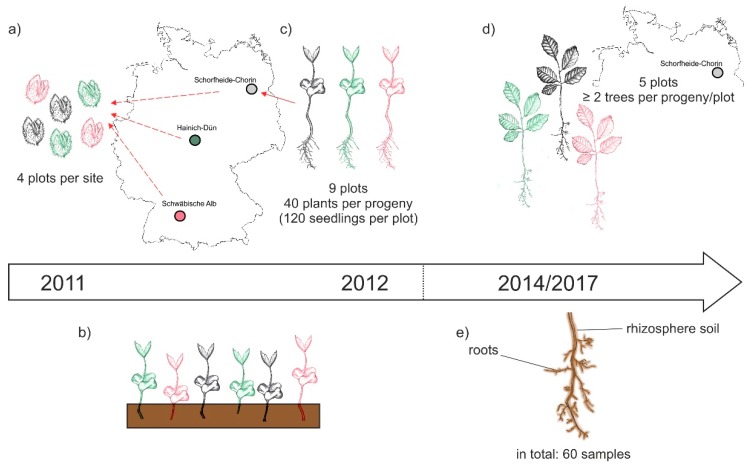
Schematic overview of the experimental design: (**a**) In October 2011 beech nuts were collected from four forest plots within the three sites of the *Biodiversity Exploratories*, i.e., Schwäbische Alb, Hainich-Dün, and Schorfheide-Chorin; (**b**) beech seedling grew in a greenhouse at Göttingen University; (**c**) in October 2012 beech seedling of different progenies were out-planted to nine forest plots per site; (**d**) in September 2014 and November 2017 beech trees on five Schorfheide-Chorin plots were sampled; (**e**) we collected roots and the attached rhizosphere soil and gained a total of 60 samples (three progenies × five plots × two sampling years × two compartments) for subsequent analyses of the beech root-associated fungal mycobiome.

The BTE was performed as part of the research within the *Biodiversity Exploratories* [32]. This large-scale project encloses 50 forest plots (100 m × 100 m) in three different sites across Germany: Schwäbische Alb (Alb) in the south west, Hainich-Dün (Hai) in central, and Schorfheide-Chorin (Sch) in the north east of the country (Figure 1a, [32]). In October 2011 beech nuts were collected on four plots of each site. Accordingly, the three different sites represent at the same time three different beech progenies. The beech nuts were stored at 0 °C and 10% moisture until January 2012, and were then germinated on moist filter paper as recently described [35]. In April 2012, the seedlings were transferred to pots (160 × 55 × 21 mm; QP 24 T/16, HerkuPlast Kubern GmbH, Germany) containing a mixture of 50% soil (Fruhstorfer Erde, Typ N, Hawita Group, Vechta, Germany), 40% coarse sand (0.7–1.2 mm), and 10% fine sand (0.4–0.8 mm). In October 2012, bare-root seedlings of similar size were selected and their mycorrhizal status was checked using a stereomicroscope (Leica DFC 420 C, Wetzlar, Germany). At the time of out-planting, no mycorrhizal structures were observed on the roots of the chosen beech plants. Forty seedlings per progeny were planted in nine plots in Schorfheide-Chorin. The progenies were randomized and planted at a 20 cm distance between plants. Due to beech mortality, only five plots were used for this study and sampled in September 2014 and November 2017. In each plot, two to five beech plants per progeny were used. A graphical overview of the collecting, cultivating and sampling procedure is given in Figure 1.

### 2.2. Soil and Root Sampling

Stems of selected trees were cut 2 cm above the soil. The soil and roots around the remaining stems were taken with a split tube sampler (diameter 12.5 cm). In an initial step, the bulk soil was carefully separated from the root system. Afterwards, the soil closely attached to the roots was removed and considered as rhizosphere soil. Fine roots were separated from the complete root system. Aliquots of rhizosphere soil and fine roots were immediately put on ice in the field and later stored at −20 °C.

### 2.3. DNA Extraction, Library Preparation, and Illumina Sequencing

We extracted the total microbial DNA from 400 mg rhizosphere soil for each sample using the Power Soil DNA Isolation Kit (Qiagen, Hilden, Germany) following the instructions by the manufacturer. Likewise, DNA from ca. 30 mg fine roots were extracted using the innu PREP-Plant DNA Kit (Analytik Jena, Jena, Germany) according to the manufacturer´s guidelines. Concentrations of DNA were determined using a NanoDrop 8000 spectrophotometer (Thermo Fisher Scientific, Dreieich, Germany). Afterwards, the DNA extracts were diluted to an equal concentration of 10–15 ng/μL.

Fungal amplicon libraries were prepares by performing amplification of the fungal ITS2 region. Thereby, DNA templates derived from fine roots sampled in 2014 were amplified using the forward primer ITS3KYO2 [36] and the reverse primer ITS4 [37], containing the Illumina adapter sequences. The PCR mixture with a total volume of 50 µL consisted of 30.5 µL sterile nuclease-free water, 10 µL 5× Phusion High-Fidelity buffer with MgCl_2_, 1 µL of 10 mM dNTPs Mix, 0.5 µL of Phusion High-Fidelity DNA polymerase (2 U/µL) (all chemicals from Thermo Fisher Scientific), 2.5 µL of each primer (10 µM) and 3 µL of DNA template. PCR reactions were performed in triplicate and with negative controls using the reaction mixture without template under the following conditions: initial denaturation for 30 s at 98 °C; 25 cycles of 98 °C for 10 s, 48 °C for 20 s and 72 °C for 20 s; final extension step of 5 min at 72 °C. PCR products were checked by agarose gel electrophoresis for appropriate size and purified using a magnetic bead-based Magsi-NGSPREP kit (Steinbrenner Laborsysteme GmbH, Wiesenbach, Germany) according to the manufacturer’s instructions. PCR products were stained using GelRed (0.01 µL/mL, GelRedTM Nucleic Acid, Biotium Inc., VWR International GmbH, Darmstadt, Germany) and visualized under ultraviolet light (Intas Science Imaging Instruments GmbH, Göttingen, Germany). Gel running conditions were 90 V for 25 min in an electrophoresis system (Power Pac 200, Biorad Laboratories Ltd., München, Germany). Purified PCR products were quantified using a Qubit dsDNA HS assay Kit in a Qubit 3.0 Fluorometer (Thermo Fischer Scientific, Dreieich, Germany) and pooled at equimolar concentrations for sequencing. Amplicons were sequenced using the dual index paired-end approach (v3, 2 × 300 bp) for the Illumina MiSeq platform. The sequencing of the root sample libraries 2014 was carried out in Göttingen (Göttingen Gemonic Laboratory).

Diluted DNA templates of the rhizosphere soil and fine roots sampled in 2017 were amplified using the primer set fITS7 [38] and ITS4 [37], containing the Illumina adapter sequences. PCR reactions with a total volume of 15 µL contained 1 µL template DNA, 0.3 µL of each primer, 7.5 µL 2× KAPA HiFi HotStar ReadyMix (Kapa Biosystems, Boston, MA, USA), and 5.9 µL nuclease free water. The PCR protocol started with an initial denaturation at 95 °C for 3 min, followed by 30 cycles of denaturation at 98 °C for 20 s, annealing at 56 °C for 20 s, elongation at 72 °C for 20 s, terminated with a final extension at 72 °C for 5 min. Thereby, each sample was amplified in triplicate, accompanied by one negative control per PCR reaction plate. PCR products were checked by gel electrophoresis, triplicates were pooled together, and purified with an Agencourt AMPure XP kit (Beckman Coulter, Krefeld, Germany). Cleaned products were then used as templates in a subsequent PCR, introducing the Illumina Nextera XT indices and sequencing adaptors according to the manufacturer’s instruction. The amplifications followed these conditions: Initial denaturation at 95 °C for 3 min, 8 cycles of denaturation at 98 °C for 30 s, annealing at 55 °C for 30 s, followed by elongation at 72 °C for 30 s, and a final extension at 72 °C for 5 min. Resulting PCR products were purified again with AMPure beads. Thereafter, the amplicon libraries were quantified using PicoGreen assays (Molecular Probes, Eugene, OR, USA) and pooled equimolar. Fragment sizes and quality of DNA sequencing libraries were checked using an Agilent 2100 Bioanalyzer (Agilent Technologies, Palo Alto, CA, USA). This final pool was used for paired-end sequencing of 2 × 300 bp with a MiSeq Reagent kit v3 on an Illumina MiSeq platform. The sequencing of rhizosphere soil 2014 and 2017 and root samples of 2017 was performed at the Department of Soil Ecology of the Helmholtz-Centre for Environmental Research—UFZ in Halle (Saale), Germany.

### 2.4. Bioinformatics

Raw forward and reverse reads were de-multiplexed by the Illumina MiSeq Reporter software package v2.5.1.3 with default parameters. Resulting fastq files, exempt of Illumina adaptors, sequencing primers and indices, were processed using the customizable pipeline DeltaMP (version 0.2; https://github.com/lentendu/DeltaMP/) on a high-performance computing cluster. We further adapted a workflow previously presented [39]: Pair-end reads with an overlap of 20–450 bp were merged using PandaSeq (version 2.11; [40]). Merged reads shorter than 120 bp or longer than 580 bp, as well as reads containing sequence ambiguities or homopolymers of 20 bp or longer were removed using MOTHUR (version 1.39.5-2; [41]). Potential chimeric sequences were discarded after using UCHIME [42] as implemented in MOTHUR [41]. Remaining sequence reads were pooled, de-replicated into unique sequences, while retaining sample-read assignment, and sorted by decreasing abundance using OBITools (version 1.2.12; [43]). Since two different primer sets, ITS3KYO2/ITS4 and fITS7/ITS4, were used for amplicon library preparation, an additional step applying ITSx (version 1.1; [44]) was necessary to extract the comparable ITS2 sequence. The retained fungal annotated reads were subsequently clustered into operational taxonomic units (OTUs) at 97% sequence similarity using VSEARCH (version 2.10.4; [45]). After removal of singletons, doubletons and tripletons, representative sequences for remaining OTUs were classified against the UNITE database (version 8.0; [46]) using the Bayesian classifier implemented in MOTHUR [41]. This fungal taxonomical information was then used to assign potential functions for each OTU using FUNGuild (version 1.1; [47]). We only considered unambiguous fungal guild classifications with the given confidence ranking “possible”, “probable”, and “highly probable”. The three identified fungal guilds were: (1) saprotrophs, (2) symbiotrophs, containing ecto-, arbuscular, ericoid, and orchid mycorrhizal fungi, as well as endophytes and lichens, and 3) pathotrophic fungi, merging parasites, pathogens and epiphytes.

The raw fungal sequences have been deposited in the National Center for Biotechnology Information (NCBI) Sequence Read Archive (SRA) under BioProject accession number PRJNA587107.

### 2.5. Data Normalization and Statistical Analyses

Analyses were performed using statistical software R [48]. Two OTU matrices representing 30 samples (5 plots × 3 beech progenies × 2 sampling years) for each compartment, roots and rhizosphere soil were used individually for subsequent analyses.

Initially, statistical differences between relative proportion of fungal phyla and guilds between rhizosphere soil and roots were evaluated using t-tests. To determine, whether beech progeny (Alb, Hai, or Sch) or sampling year (2014 or 2017) affected rhizosphere or root fungal OTU richness two-way analyses or variance (ANOVA) were performed. Likewise, the impact of beech progeny and sampling year was tested on the fungal community composition. For this, sequence abundance within the OTU matrices were transformed into presence-absence data. Afterwards, the “adonis” command of vegan [49] was applied to perform permutational multivariate analyses of variance (perMANOVA) with 999 permutations. The overall β-diversity, considering fungal turnover, i.e., fungal OTU replacements between 2014 and 2017, and nestedness, i.e., gain and loss of OTUs from one sampling year to the other was assessed using the “beta.temp” command of the betapart R package [50]. The statistical difference between the β-diversity of rhizosphere soil and roots was also evaluated using a *t*-test. To visualize the proportional fluctuation of fungal OTUs from 2014 and 2017 across the different progenies, we used the R package riverplot [51].

## 3. Results

### 3.1. Sequence Data at a Glance

From 30 rhizosphere soil and 30 root samples taken at two time points (2014 and 2017) from five plots in the *Exploratory* Schorfheide-Chorin of three different beech progenies (Alb, Hai, and Sch), a total of 5,995,978 abundant fungal sequences were obtained, which clustered into 3807 fungal OTUs. Thereof, 3536 fungal OTUs was found in the rhizosphere soil and 2350 fungal OTUs were associated to beech roots. The proportional taxonomical and guild-based (i.e., saprotrophs, symbiotrophs, or pathotrophs) distribution within shared, rhizosphere-, and root-specific fungal OTU composition is summarized in Table 1. The majority of fungal OTUs was shared between rhizosphere soil and roots. Moreover, the fungal composition was dominated by ascomycotous saprotrophs.

**Figure 2:**
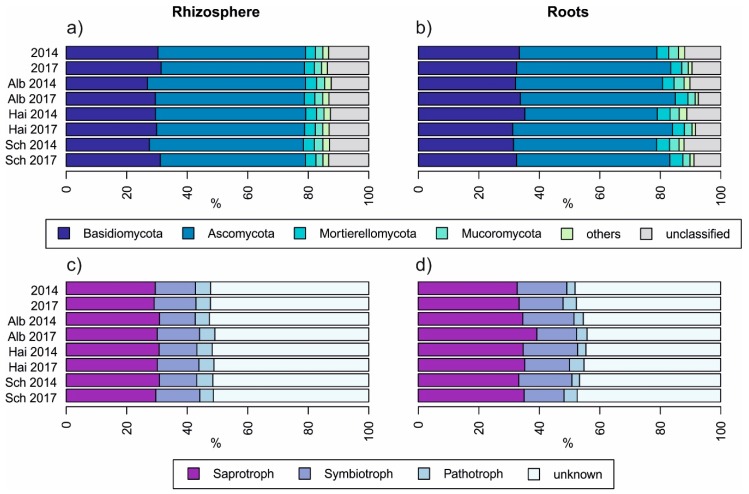
Overview of relative fungal taxonomical (**a**,**b**) and guild-based (**c**,**d**) distribution amongst beech progenies and sampling years, Alb—Schwäbische Alb, Hai—Hainich-Dün or Sch—Schorfheide-Chorin.

As displayed in Figure 2, neither sampling years nor between beech progenies, fungal taxonomy and guilds differed strongly. Comparing the fungal taxonomy of rhizosphere soil with those of roots (Figure 2a,b) we found that the relative percentage of Basidiomycota was higher in roots than in the rhizosphere soil (*p* < 0.001) and accordingly, there was a smaller relative percentage of unclassified fungi in roots (*p* < 0.001). This further resulted in a higher relative percentage of symbiotrophs and saprotrophs in beech roots than in the rhizosphere soil (*p* < 0.001 in both cases, Figure 2d). The proportion of pathogens was higher and less variant in rhizosphere soils than in roots (*p* < 0.001, Figure 2c).

### 3.2. Beech Progeny without Impact on Temporal Development on Fungal Diversity

We applied two-way ANOVA to test whether beech progeny or sampling year had an impact on total fungal OTU richness or OTU richness of different guilds (i.e., saprotrophs, symbiotrophs, or pathotrophs) in rhizosphere soil or roots. There was no effect of beech progeny on OTU richness in any of the different fungal guilds (Table 2). There was also not effect of sampling year on the fungal OTU richness within the rhizosphere soil. In contrast, the richness of total, saprotrophic, and pathotrophic fungal OTUs in roots was significantly different between the sampling years 2014 and 2017.

### 3.3. Beech Progeny without Impact on Temporal Development on Fungal Community Composition

Likewise, we tested the impact of beech progeny and sampling year on the total, saprotrophic, symbiotrophic, and pathotrophic fungal community composition using perMANOVA. No effect of beech progeny of fungal communities neither in rhizosphere soil nor in roots could be detected (Table 3). Accordingly, a HFA of beeches original from Schorfheide-Chorin could be excluded. However, all root fungal communities changed significantly in composition from 2014 to 2017. The same applied for fungal communities of the rhizosphere, with exception of the symbiotrophs.

### 3.4. Fungal Community Turnover Independent of Beech Progeny

**Figure 3:**
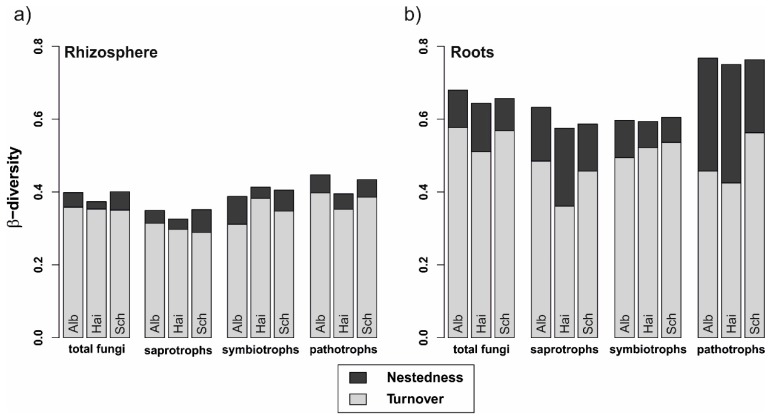
Variability of total fungal, saprotrophic, symbiotrophic, and pathotrophic community assemblages in (**a**) rhizosphere and (**b**) beech roots from 2014 to 2017. Stacked bars represent overall beta-diversity (β_SOR_) for the three beech progenies (Alb—Schwäbische Alb, Hai—Hainich-Dün or Sch—Schorfheide-Chorin), computed using the R package betapart [50], light grey sections of the bars represent the contribution of the fungal turnover (β_SIM_) and dark grey sections account for the fungal nestedness (β_SNE_).

The fungal β-diversity was higher in roots than in the rhizosphere soil (*p* < 0.001 in all cases, Figure 3). Similarly, this difference accounted for all fungal guilds, regardless of beech progeny. However, the pathotrophs in the roots showed the highest β-diversity. Besides, the overall β-diversity was divided into turnover, i.e., fungal OTU replacements between 2014 and 2017, and nestedness, i.e., gain and loss of OTUs from one sampling year to the other. In general, the turnover had a higher proportion than the nestedness, which was overall low for fungi in the rhizosphere and increased for fungi in the roots.

**Figure 4:**
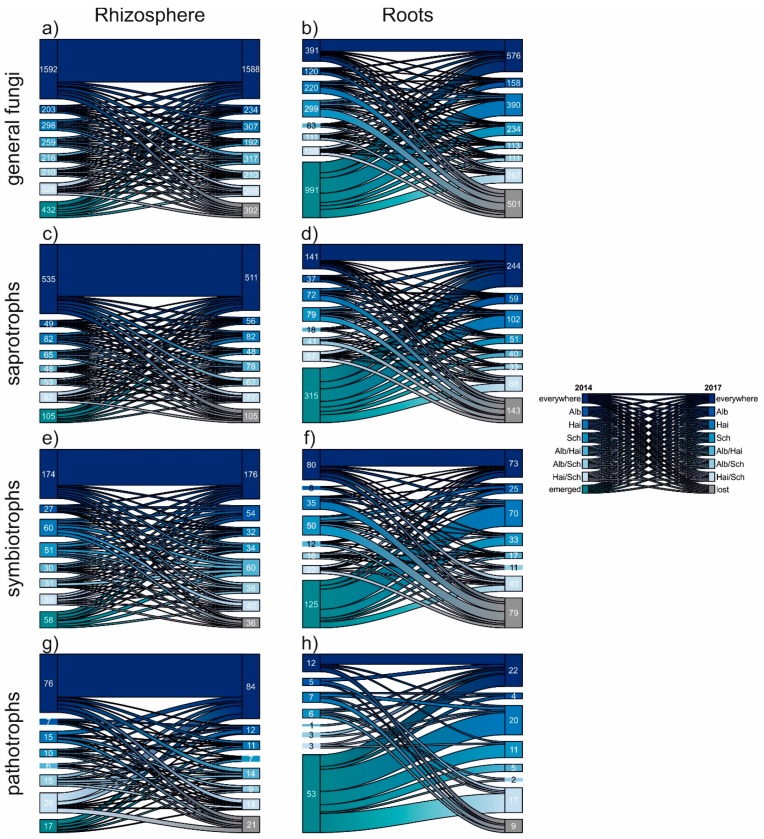
Riverplots displaying the temporal development of general (**a**,**b**), saprotrophic (**c**,**d**), symbiotrophic (**e**,**f**) and pathotrophic (**g**,**h**) fungal OTUs from 2014 to 2017. The individual plots show in detail the proportions of OTU occurrences within the examined samples: beech progenies: Alb—Schwäbische Alb, Hai—Hainich-Dün or Sch—Schorfheide-Chorin, emerged OTUs did not appear in 2014, but in 2017. The contrary is the case for lost OTUs, which occurred in 2014, but no longer in 2017.

The fluctuation of the fungal OTUs was unravelled in detail using riverplots (Figure 4). Although the different fungal communties in rhizosphere and roots were composite of different amounts of divergent OTUs, this vizualisation method allowed a proportional comparison. Again independently of beech progeny, the fungal communities of the rhizosphere were rather composed of generalists, which occured everywhere in both sampling years. In contrast, root fungi were characterized, as already mentioned, by a high nestedness. This is supported by the particullarly high proportion of emerging OTUs, which did not occur in 2014, but in 2017. Also the relative proportion of fungal OTUs lost in 2017 was bigger for roots. However, we noticed that the proportion of general, saprotrophic and pathogenic fungal OTUs occuring in roots of all progenies (i.e., everywhere) was increased 2017. This hints towards a slow homogenization amongst the mentioned fungal groups.

## 4. Discussion

The BTE aimed to understand, if and how early stage root-associated mycobiomes are affected by beech progeny over time. Similarly, we wanted to know, if the beeches originating from our study site Schorfheide-Chorin displayed a HFA by having an enhanced proportion of symbionts coupled with a decreased number of pathotrophs in their root-associated mycobiome. For this, we analyzed the fungal communities in rhizosphere soil and in roots of beeches of three different progenies, which can be considered as different beech ecotypes [34]. The overall diversity was high with more than 3000 fungal OTUs. Thereby, the fungal OTU richness was higher in the rhizosphere soil than in the beech roots. Most root fungi are recruited from the surrounding rhizosphere soil and, thus only a small proportion of unique root fungi were detected, which is in accordance with previous studies [52,53,54].

Our results did not support our first hypothesis that the progeny impacts on the mycobiome of beech roots. Neither fungal OTU richness, fungal community structure, taxonomic nor functional composition were significantly impacted by beech progeny. Previously, Perez-Izquierdo and colleagues [19] found that the genotypes of *Pinus pinaster* is clearly structuring the associated fungal communities. Similar impacts on fungal communities were found for different genotypes of the broadleaf *Populus* [18] and varying phenotypes of *Abies alba* [17]. In contrast to the mentioned studies [17,18,19], which used trees of higher ages and bigger diameter at breast height, the beeches in our study were very young, just five years at the second sampling in 2017. A comparable approach carried out in a pot experiment led to the same absent progeny effect on EMF communities [35]. These related results might indicate that different ecotypes deploy their influence on the mycobiome only at a later tree development stage or indeed not at all in the case of beech. In fact, previous investigations on mature beeches showed no impact of their genotypes on fungal composition [55]. However, abiotic parameters are probably involved in shaping the fungal communities at early stages. It is well known that particular physico-chemical soil properties are of importance for shaping fungal soil and root community patterns [52]. The initial growth of the beeches within BTE took place in the greenhouse in neutral soil, to avoid any kind of advantage back in the field situations. Consequently, the mycobiome of all beeches, independent of progeny, had to deal with comparable soil conditions, characterized by low pH and high C/N ratios [33]. Apparently, site specific properties led to similar recruiting of saprotrophic, symbiotrophic, and parasitic fungi in rhizosphere and roots. Hence, no HFA for the mycobiome of beeches original from Schorfheide-Chorin was detected, which disagrees with our second hypothesis.

It was recently shown that temporal changes are equivalent to large-scale distance decay patterns concerning changes in fungal communities [14,56]. Our study adds to this limited knowledge of fungal development over time by displaying differences of fungal richness and community structure in rhizosphere soil and roots. Although the fungal OTU richness for rhizosphere soil did not differ between the sampling years, an enhanced number of general, saprotrophic, and pathotrophic root fungal OTUs was detectable in 2017. Root growth over time increase the root surface and offers more fungal colonization space [16]. In our study, this suggested mechanism was not true for the OTU richness of symbiotrophs, i.e., EMF, which did not increase, though the emerged symbiotrophic OTUs (i.e. not present in 2014) exceeded the number of lost symbiotrophic OTUs (not present in 2017) in roots. This pinpoints that although beech roots potentially grew, the plant-derived carbon is invested in different rather than in more EMF. This is supported by the fact that the root symbiotrophic fungal communities did not change significantly from 2014 to 2017. EMF can be differentiated into “early (successional) stage” to “later stage” taxa [57,58]. “Early stage” EMF develop themselves from spores and fragmented mycelia and need only little initial nutrient supply for root colonization [57,58]. Experiments show that the success and the performance of colonization of EMF with sterile-grown tree seedlings is improved, if they are planted in mature forests with hyphae associated with living roots (see review by Jones et al., 2003 [57]). Moreover, ectomycorrhizal colonization is characterized by priority effects, i.e., early colonizers can outcompete others when new root tips become available [59]. Thus, some of the these ectomycorrhizal pioneers can persist many years even after new tree transplantation events [60], which might account for the stable part of the symbiotrophic communities. Supposedly, saprotrophic fungi benefit from this relative stability of symbiotrophic diversity and community structure. Especially from older forest stands it is known that some EMF, which are enzymatically equipped for decomposition processes, compete with saprotrophic fungi [61,62]. In mature forests this then results in declining relative abundances of saprotrophs due to increased impact of EMF [13,63]. Apparently, the “early stage” EMF do not possess the needed enzymes. Comparable to the results by Awad et al. [30], we hence found saprotrophs to be the most abundant fungal group in rhizosphere soil and roots, which suggests that they can fulfill and develop their fundamental niche without or only with little competition of EMF at these early stages of beech development.

Pathotrophic fungi made up the smallest proportion of the overall fungal community, but displayed the highest β-diversity, with around 40% within rhizosphere and up to 75% community change within roots from 2014 to 2017. These results show that young beeches face multiple potentially harmful fungi, which are either plant or even beech pathogens. Alternatively, the full microbiome of the tree prohibited tree mortality or any phenotypical signs of tree infections by this fungal guild [64,65]. However, from our experimental approach it is impossible to tell what drove the pathotrophic fungal communities in particular. It was recently shown that shifts in fungal composition with increasing forest age alters enzymatic profiles and available nutrients, which again impact the whole mycobiome [13]. Thus, the temporal divergent fungal pathotrophs could just be a consequence of this feedback loop.

## 5. Conclusions

Our study revealed a high temporal variability of the root-associated mycobiome of young beech trees. Independently of beech progeny, the fungal communities in rhizosphere soil and roots displayed high β-diversity, whereby the rhizosphere communities served as pool for displayed temporal shifts of the root fungal communities. Hence, our results add to the general knowledge on early-stage tree mycobiomes. Possible HFA and general progeny effects could be tackled by a longer exposition time of beech trees in the field. Moreover, our study considered only one site and comparable experiments should be repeated elsewhere to verify the found fungal assembly patterns over time. Likewise, the EMF communities of mature trees should be analyzed in future studies to determine potential sources of succession processes.

## Figures and Tables

**Table 1:** Overview of fungal operational taxonomic unit (OTU) distribution for shared, rhizosphere, and root fungal composition. Proportion refers to 3807 OTUs as 100 %.

	Number of Shared OTUs	Number of Rhizosphere OTUs	Number of Root OTUs
**Total**	2079 (54.61%)	1457 (38.27%)	271 (7.12%)
Ascomycota	1014 (26.63%)	673 (17.68%)	101 (2.65%)
Saprotroph	373 (9.80%)	207 (5.44%)	63 (1.65%)
Symbiotroph	136 (3.57%)	74 (1.94%)	12 (0.32%)
Pathotroph	58 (1.52%)	60 (1.58%)	2 (0.05%)
Unknown	447 (11.74%)	332 (8.72%)	24 (0.63%)
Basidiomycota	665 (17.47%)	454 (11.93%)	139 (3.65%)
Saprotroph	153 (4.02%)	114 (2.99%)	52 (1.37%)
Symbiotroph	146 (3.84%)	100 (2.63%)	48 (1.26%)
Pathotroph	24 (0.63%)	19 (0.50%)	2 (0.05%)
Unknown	342 (8.98%)	221 (5.81%)	37 (0.97%)
Other phyla	400 (10.51%)	330 (8.66%)	31 (0.82%)
Saprotroph	118 (3.10%)	54 (1.42%)	1 (0.03%)
Symbiotroph	6 (0.16%)	8 (0.21%)	0 (0.00%)
Pathotroph	4 (0.11%)	7 (0.18%)	0 (0.00%)
Unknown	272 (7.14%)	261 (6.85%)	30 (0.79%)

**Table 2:** Impact of beech progeny and sampling year on OTU richness of general fungal, saprotrophic, symbiotrophic and pathotrophic in rhizosphere and roots assessed by ANOVA, in bold significant effects.

	Rhizosphere				Roots			
	Beech Progeny		Sampling Year		Beech Progeny		Sampling Year	
	F	p	F	p	F	p	F	p
Richness total fungi	1.904	0.169	0.075	0.786	2.217	0.129	6.304	**0.019**
Richness saprotrophs	1.815	0.183	0.105	0.749	2.020	0.153	11.443	**0.002**
Richness symbiotrophs	0.361	0.701	0.216	0.646	2.081	0.145	0.948	0.339
Richness pathotrophs	0.803	0.459	0.861	0.362	2.846	0.076	20.421	**<0.001**

**Table 3:** Impact of beech progeny and sampling year community composition of general fungi, saprotrophs, symbiotrophs, and pathotrophs in rhizosphere and roots assessed by perMANOVA, in bold significant effects.

	Rhizosphere				Roots			
	Beech progeny		Sampling year		Beech progeny		Sampling year	
	R²	p	R²	p	R²	p	R²	p
Total fungi	0.030	1.000	0.081	**0.014**	0.053	0.688	0.160	**0.001**
Saprotrophs	0.029	1.000	0.090	**0.005**	0.053	0.618	0.156	**0.001**
Symbiotrophs	0.034	0.099	0.047	0.227	0.053	0.702	0.126	**0.001**
Pathotrophs	0.033	0.994	0.084	**0.003**	0.050	0.677	0.160	**0.001**
